# Using Daily Stretching to Counteract Performance Decreases as a Result of Reduced Physical Activity—A Controlled Trial

**DOI:** 10.3390/ijerph192315571

**Published:** 2022-11-23

**Authors:** Konstantin Warneke, Andreas Konrad, Michael Keiner, Astrid Zech, Masatoshi Nakamura, Martin Hillebrecht, David G. Behm

**Affiliations:** 1 Institute for Exercise, Sport and Health, Leuphana University, 21335 Lüneburg, Germany; 2 School of Human Kinetics and Recreation, Memorial University of Newfoundland, St. John’s, NL A1C 5S7, Canada; 3 Institute of Human Movement Science, Sport and Health, University of Graz, 8010 Graz, Austria; 4 Department of Sport Science, German University of Health & Sport, 10587 Ismaning, Germany; 5 Department of Human Motion Science and Exercise Physiology, Friedrich Schiller University, 07743 Jena, Germany; 6 Faculty of Rehabilitation Sciences, Nishi Kyushu University, Ozaki, Kanzaki, Saga 842-8585, Japan; 7 University Sports Centre, University of Oldenburg, 26129 Oldenburg, Germany

**Keywords:** jump performance, flexibility, maximum strength, stretching, physical activity

## Abstract

There are many reasons for reduced physical activity leading to reduced maximal strength and sport-specific performance, such as jumping performance. These include pandemic lockdowns, serious injury, or prolonged sitting in daily work life. Consequently, such circumstances can contribute to increased morbidity and reduced physical performance. Therefore, a demand for space-saving and home-based training routines to counteract decreases in physical performance is suggested in the literature. This study aimed to investigate the possibility of using daily static stretching using a stretching board to counteract inactivity-related decreases in performance. Thirty-five (35) participants were either allocated to an intervention group (IG), performing a daily ten-minute stretch training combined with reduced physical activity or a reduced physical activity-only group (rPA). The effects on maximal voluntary contraction, range of motion using the knee-to-wall test, countermovement jump height (CMJ_height_), squat jump height (SJ_height_), drop jump height (DJ_height_), contact time (DJ_ct_) and the reactive strength index (DJ_RSI_) were evaluated using a pre-test-post-test design. The rPA group reported reduced physical activity because of lockdown. Results showed significant decreases in flexibility and jump performance (d = −0.11–−0.36, *p* = 0.004–0.046) within the six weeks intervention period with the rPA group. In contrast, the IG showed significant increases in MVC90 (d = 0.3, *p* < 0.001) and ROM (d = 0.44, *p* < 0.001) with significant improvements in SJ_height_ (d = 0.14, *p* = 0.002), while no change was measured for CMJ_height_ and DJ performance. Hence, 10 min of daily stretching seems to be sufficient to counteract inactivity-related performance decreases in young and healthy participants.

## 1. Introduction

Physical inactivity is a consistent part of many people’s daily life [1], as well as the possibility for prolonged phases of immobilization [2,3,4], which can have significant negative influences on metabolic and musculoskeletal health [5,6] in sports performance. In addition to injuries and frequent sitting, evidence highlights significantly decreased physical activity and motivation to train [7] in the majority of people due to the COVID-19 lockdown(s) [8,9,10]. As a consequence, reduced physical fitness, including strength capacity and maximal strength (MSt), were reported. Restoring physical fitness after times of prolonged immobilization is of high importance, and this is the aim of several rehabilitation programs to avoid all causes of mortality [11]. Therefore, Schwendiger & Pocecco [11] provided some recommendations for home-based training programs, which should include exercise for the cardiorespiratory system and the musculature, stating the demand for safe, space-saving and efficient training programs, which can be performed without heavy equipment as a home-based training routine [11,12,13].

It is well known that performing resistance training is linked with improvements in MSt [14,15,16], muscle hypertrophy [14,17,18] and sport-specific performance, such as jumping and sprinting [19,20,21,22]. However, there are limited possibilities for using heavy weights in a home-based training program during a period of inactivity or immediately after injury; therefore, alternative training methods are required to counteract MSt impairments. Fortunately, current literature points out the use of high-volume stretch training to improve MSt (29%; d = 1.24), muscle thickness (MTh) (15.3%; d = 0.84) and range of motion (ROM) (27.3%; d = 0.87) using two to seven sessions per week with stretching durations of up to two hours per day for six to ten weeks [23,24,25,26,27,28,29]. In contrast to the highly effective improvements in MSt, MTh and ROM when using two hours of daily stretching, Yahata et al. [23] were also able to show significant (6% (d = 0.35)) increases in plantar flexor MSt using stretching for durations of only 30 min (6 × 5 min) two days per week using a stretching board. Furthermore, Shrier [30] and Medeiros & Lima [31] reviewed the available literature illustrating the benefits of stretching routines for physical performance, such as jumping and sprinting. However, there are only a few studies investigating the effects of long-term stretching routines on speed–strength performance, such as jumping or sprinting. Panidi et al. [32] were able to point out significant increases in one-leg countermovement jump height (CMJ_height_) (27.3 ± 30%) in response to 12 weeks of stretch training, using six different exercises, including the stretching board. However, the participants were young female volleyball players; consequently, the stretch training was accompanied by regular volleyball training. Furthermore, although the contralateral limb served as a control (i.e., a possibility of cross-education effects), no regular control group was included in the study design. Kokkonen et al. [33] showed that stretching of the lower extremity using three × 15 s with about 15 stretching exercises 3 days per week with a weekly volume of 120 min increased the jumping height by 3.9% (d = 0.14), and the jumping distance in the standing long-jump by 2.2% (d = 0.11). While significant, both increases were of trivial magnitudes for an effect. Bazett-Jones et al. [34] could not detect any significant changes in jumping and sprinting performance in response to stretching 4 × 30 s, 3 days per week using track and field female athletes. Also, Nakamura et al. [35] were not able to detect any improvement in drop jump (DJ) height in response to 3 × 30 s stretching, 3 days per week. These conflicts in the literature suggest that the influence of stretch-induced MSt increases on jumping performance seems to be controversial. Thus, considering the high relevance of maximal strength and jumping performance in many sports, the effects of using stretch training to improve those parameters need to be extended. Since immobilization-induced atrophy and performance losses are very common due to injury, it is hypothesized that prolonged stretch training could be used as a method accessible to everyone to counteract performance losses, even during phases of reduced physical activity during pandemic lockdowns.

Therefore, the aim of this study is to investigate the influence of daily stretch training in a usually physically active population undergoing a phase of inactivity due to the COVID lockdown using a stretching board on maximal strength, flexibility, and jumping performance.

## 2. Materials and Methods

To evaluate the effects of stretching on muscular performance, healthy physical education students were recruited from the local university to perform a daily six-week stretching intervention. Using a pre-test and post-test design, MSt and ROM in the plantar flexors, as well as jumping performance using the countermovement jump (CMJ), squat jump (SJ) and the DJ, were investigated. 

### 2.1. Participants

Based on the literature, moderate to high size effects (Warneke et al., 2022) were assumed for sample size calculation. G-Power analysis using d = 0.7, two groups and two measurements showed a total sample size of at least 30 participants. Thirty-five (35) healthy male and female students from physical education programs were divided into intervention (IG) and reduced physical activity groups (rPA) based on their willingness to participate. The subject description is shown in Table 1. Reduced physical activity was assumed in all the participants included because of COVID-19 restrictions, as gyms and universities stayed closed after the exams finished. Before the lockdown-induced inactivity period, participants regularly practiced in team sports, gymnastics or track and field classes with a moderate training level (participating in 2–3 sports classes within the university sports program per week). Thus, it can be assumed that participants in the rPA group showed reduced physical activity compared to the phase before exams, where they regularly trained on the University sports courses. Within the intervention period, no additional (resistance) training was performed by the rPA group, and the University sports program was closed. Participants with self-reported significant injury within the last six months leading to a prolonged phase of immobilization and participating in a rehabilitation training program were excluded from the study. Each subject was informed of the experimental risks involved with the research. All subjects provided written informed consent to participate in this study. Approval for this study was obtained from the ethical review board at the University of Oldenburg (No. 121-2021). The study was performed with the use of human subjects in accordance with the Helsinki Declaration.

### 2.2. Testing Procedure

The maximum isometric strength in a 90° knee joint angle (MVC90) was recorded for both legs using bilateral maximum strength testing. The measurement procedure was performed as previously described in Warneke et al. [27]. For this purpose, the subject was instructed to perform plantar flexion for three seconds with the maximum possible force in response to an acoustic signal to press against the pad of the measuring device. The calf muscle testing device was set to a 90° angle with the subject’s ankle and knee joints. Testing was performed until the achieved force values stopped increasing, with a minimum of five trials. The maximum force was determined in each case using a 10 × 10 cm force measurement platform in which force sensors “Kistler Element 9251A”, with a resolution of 1.25 N, a pull-in frequency of 1000 Hertz, and a measurement range of ±5000 N, were installed. The vertical forces (Fz) were recorded. A charge amplifier, “Typ5009 Charge Amplifier”, and a 13-bit analog-to-digital converter NI6009 were used. The reliability can be classified as high with ICC = 0.994 [27].

### 2.3. ROM Measurement

**Figure 1:**
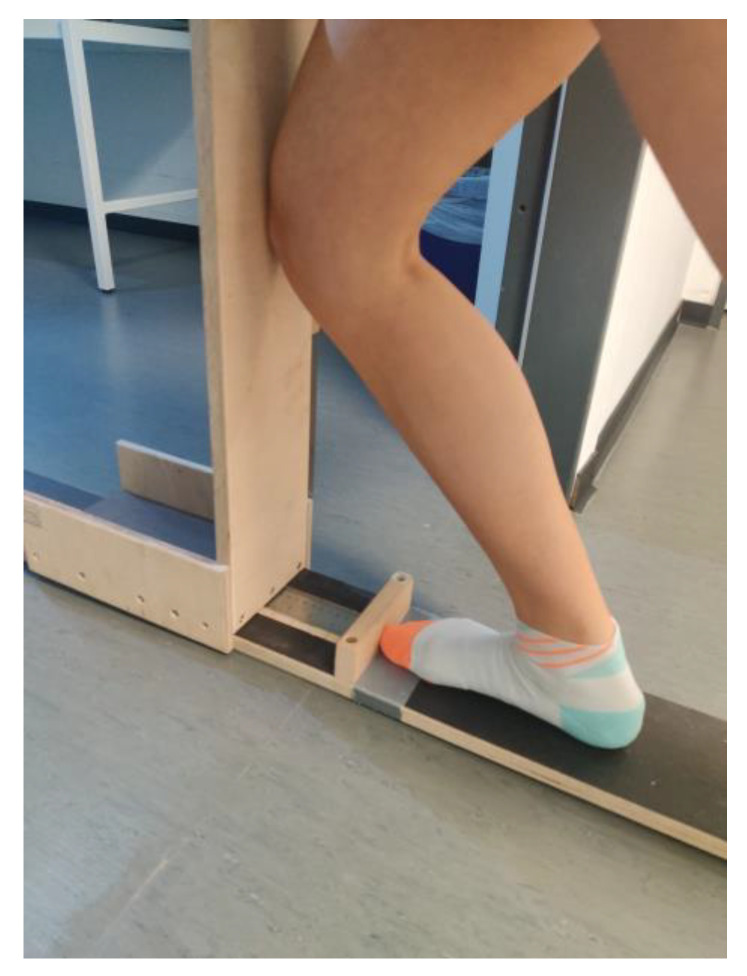
Using the knee-to-wall test to measure the range of motion in the upper ankle joint.

The ROM in the ankle joint was recorded in IG and rPA via the “knee-to-wall test” (KtW), as previously described in Warneke et al. [28]. A sliding device was used for the KtW. The subject was instructed to place a foot on the attached marker. The contralateral leg was held in the air, and the subject could hold onto the wall with their hands. To record the range of motion, the subject pushed the board of the sliding device forward until the heel of the standing leg lifted off. For this purpose, the investigator pulled on a sheet of paper placed under the subject’s heel. The measurement was finished as soon as this could be removed. The mobility was read in cm from the attached measuring tape (see Figure 1). Three valid trials were performed per leg, and the maximum value was used for evaluation. The reliability of the measurement can be considered high, with an ICC of 0.987 and 0.992 [28].

### 2.4. Jumping Measurement

Jump performance testing was performed using the three standard jump tests. First SJ, then CMJ were measured (5 trials each, with a 1-min rest between jumps) using a force plate (BP600900-4000, AMTI, Watertown, MA). The jump height was determined by the flight time in all three jump forms. The maximum jump height achieved was included for further calculation. The jumps were performed with the hands fixed on the hips. The SJ was initiated from a squat position (approx. 90° knee angle) after a 2-s hold without momentum. The CMJ was initiated from an upright position to utilize the momentum of a preceding squat movement (to approx. 90° knee angle) in the actual jump. The test–retest reliability is reported for CMJ and SJ height (CMJ_height_/SJ_height_) ICC = 0.94 [21]. The DJ test was carried out from a 24 cm drop height. DJs were also measured (5 trials each). With an initial step, subjects “fell” from a box (of corresponding height) and were instructed to jump as high as possible after both feet had contacted the ground. The hands were also fixed on the hips. They were further encouraged to reduce ground contact time to a minimum with a maximum jumping height. Shorter durations of ground contact and higher jumps reflect better reactive power. The reactive strength index (RSI) was calculated from this data (RSI = jump height/contact time in m/s). The participants paused for 1 min between jumps and 5 min between different jump heights. The test–retest reliability of DJ is reported between ICC = 0.85–0.88 [36].

### 2.5. Intervention

**Figure 2:**
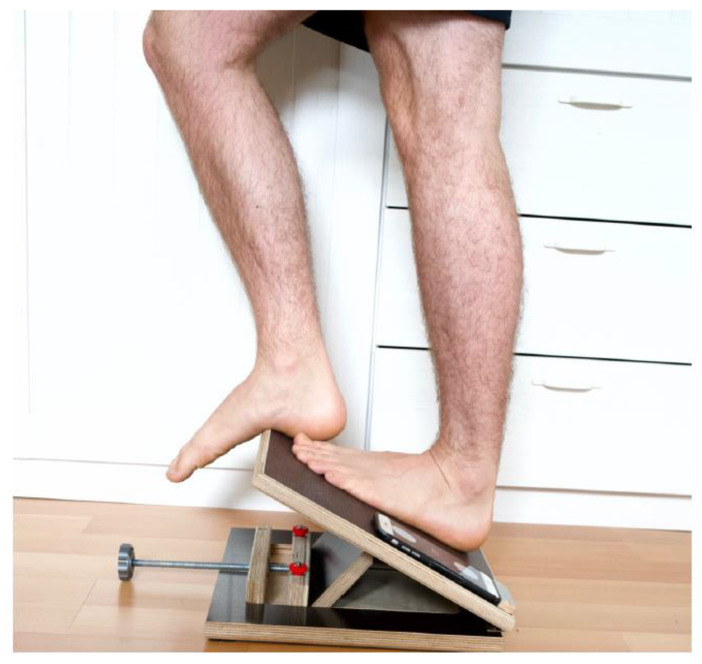
Using a stretching board for stretching the plantar flexors.

The intervention consisted of daily stretching of the plantar flexors for 10 min per leg for six weeks. The training was performed using a stretching board (see Figure 2) comparable to that used by Yahata et al. [23] and Cé et al. [37]. The participants were instructed to set the angle of the board to reach a maximum stretching stimulus, with a high stretching pain of 7–8 out of 10 on a numeric pain scale, using a subjective stretching pain as previously mentioned by Nakamura et al. [38]. The stretching was performed unilaterally, but for both legs.

### 2.6. Data Analysis

The data analysis was performed with SPSS 28. Data are provided using means (M) ± standard deviation (SD). Normal distribution was given (Shapiro Wilk test). Reliability was tested and is supplied with intraclass correlation coefficients (ICC) and a coefficient of variability (CV), including a 95% confidence interval (95% CI) for assessments which are listed in Table 2. Moreover, the Levene test for homogeneity in variance was performed. The *t*-Test for independent samples was used to evaluate differences in pre-test values. A mixed model analysis of variance was performed to investigate the time and the time*group interaction effect. Effect sizes are presented as Eta squares (ƞ^2^) and categorized as: small effect ƞ^2^ < 0.06, medium effect ƞ^2^ = 0.06–0.14, large effect ƞ^2^ > 0.14 [39]. Additionally, to assess whether there were specific post hoc significant increases in IG or decreases in rPA, paired *t*-tests were used. To adjust the α-error, the false discovery rate with the Benjamini-Hochberg method was used [40]. From this, Cohen’s d [39] effect sizes are reported and categorized as: trivial d < 0.2, small d < 0.5, medium d = 0.5–0.8, and large effects d > 0.8. Furthermore, correlation coefficients were calculated between MSt and jumping performance. Post hoc power analysis, using the G*Power software package (version 3.1.4, HHU Düsseldorf, Germany), was performed.

## 3. Results

**Figure 3:**
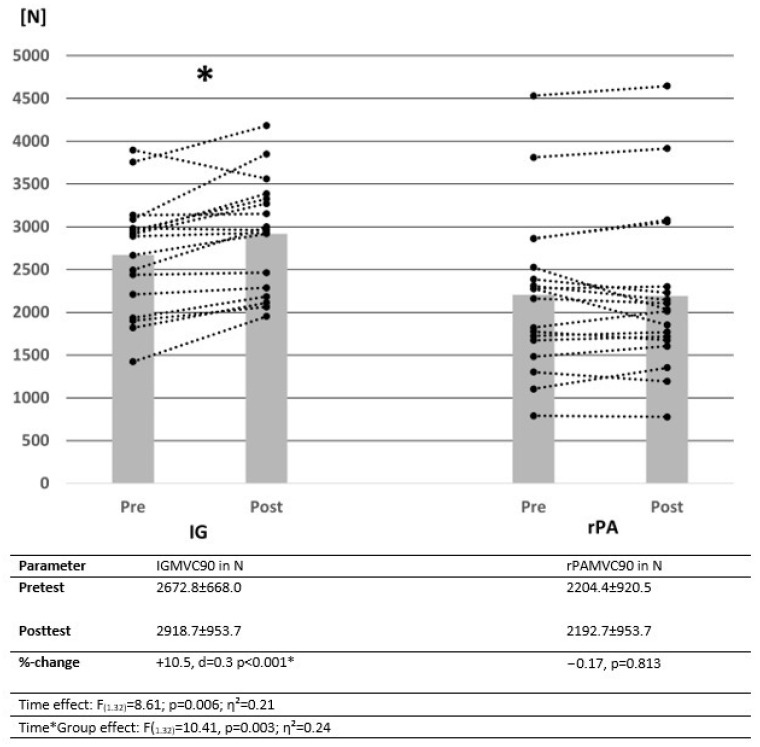
Change in mean values and individual cases of maximal voluntary contraction of control and intervention groups over time. Data presented as means ± SD. IG = intervention group, rPA = reduced physical activity group, MVC90 = maximal voluntary contraction in the plantar flexors with 90° knee joint angle, * = significantly different from pre-test to post-test.

**Figure 4:**
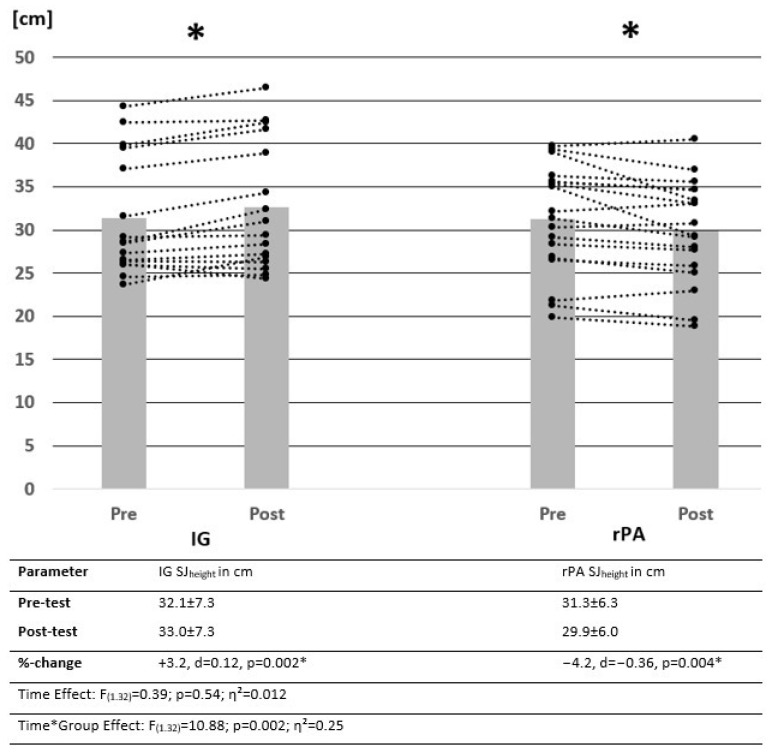
Change in mean values and individual cases of squat jump of control and intervention groups over time. Data presented as means ± SD. IG = intervention group, rPA = reduced physical activity group, SJ = squat jump, * = significantly different from pre- to post-test.

**Figure 5:**
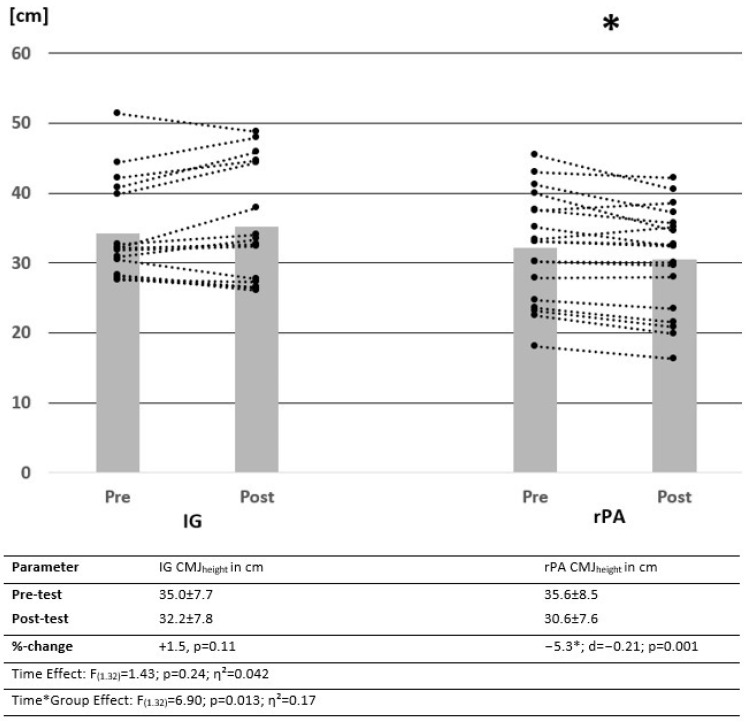
Change in mean values and individual cases of countermovement jump of control and intervention groups over time. Data presented as means ± SD. IG = intervention group, rPA = reduced physical activity group, CMJ = countermovement jump, * = significantly different from pre- to post-test.

All participants completed the intervention. There were no significant group differences in the pre-test values, with *p* = 0.096–0.963 for MSt, ROM and jumping height. Changes in MSt and jumping height measured via SJ_height_ and CMJ_height_ from pre-test to post-test are illustrated by descriptive data, as well as the results of the ANOVA in Figure 3, Figure 4 and Figure 5. Power analysis performed with G Power showed a power of β − 1 = 78.7% for interaction effects.

### 3.1. Evaluation of Maximal Strength in the Plantar Flexors

The results show significant increases in MVC90, with a significant time effect, as well as a significant interaction effect. There was a significant pre- to post-test force increase in IG, while no significant change could be determined in rPA.

### 3.2. Evaluation of Jumping Height Using the Squat- and Counter Movement Jump

Results showed no significant time effect but significant interaction effects in SJ_height_ and CMJ_height_. While there was a significant increase in SJ height in the IG, the rPA group showed a significant reduction in jumping height. For the CMJ, there was a significant decrease in CMJ_height_ without any significant change in the IG.

### 3.3. Evaluation of Jumping Performance Using the Drop Jump

**Figure 6:**
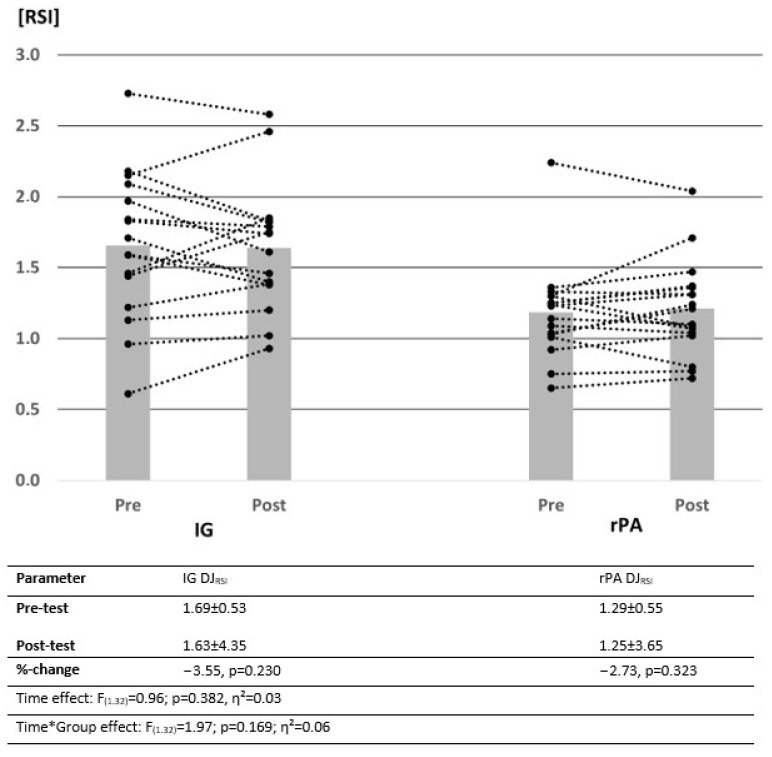
Change in mean values and individual cases of reactive strength index of control and intervention groups over time. Data presented as means ± SD. IG = intervention group, CG = control group, DJ_RSI_ = reactive strength index.

Since significant pre-test differences were present between the IG and rPA for the contact time and the RSI, evaluation for those parameters was performed after transforming the pre-test values to 100%. Therefore, differences between IG and rPA were calculated by percentage increases. Changes in RSI are illustrated in Figure 6. Values of DJ_height_ and the contact time of the drop jump are provided in Table 3. In the drop jump, no statistically significant changes from the pre-test to the post-test could be detected.

### 3.4. Evaluation of Flexibility Using the Knee-to-Wall Test

In the IG, there were significant time and time × group interaction effects in the KtW for both legs (Table 4). While no significant changes were observed in the rPA, there were significant increases in ROM in IG for both legs. 

## 4. Discussion

The major findings of the present study demonstrated significant decreases in flexibility and jump performance within the six-week intervention period in the rPA group, while IG showed significant, small-magnitude increases in MSt, and flexibility, whereas the significant increase in jumping performance was of a trivial magnitude. From these results, it can be inferred that 10 min of daily stretch seems to be sufficient to counteract inactivity-related performance decrease in healthy participants.

The results of this study are in accordance with previous literature showing increases in MSt and ROM [23,24,25,26,33] and increases in jumping performance [32,33] due to long-term stretching interventions. It is well-accepted that stretch training leads to significant increases in flexibility with a dose–response relationship [41,42,43]. Small significant increases in MSt obtained in the present study confirm the increases of 6.9% (d = 0.35) from Yahata et al. [23] using a similar weekly stretching volume of 60 min per week. However, they applied only two sessions per week with longer stretching durations of 30 min. It is speculated that stretch-induced increases in MSt can be attributed to mechanical tension if performed with sufficient volume and intensity [27,28]. As a general transferability of MSt to speed strength has been reported [44], leading to increases in sport-specific movements such as jumping and sprinting [19,20,22], it was hypothesized that increases in MSt due to stretching could, on the one hand, lead to significant improvements in speed strength, resulting in increased jumping performance. On the other hand, studies performed with animal models showed an increase in ST-fibers, accompanied by a decrease in FT fibers, leading to a decreased contraction velocity after a chronic stretch training intervention [45,46]. From this data, a decremental influence on jumping performance could be speculated. 

This study confirmed reductions in physical fitness parameters due to reduced physical activity [8,9,47], as participants were not allowed to perform regular training within the intervention, with the exception of daily stretching (IG). While there were significant increases in performance with MVC90, KtW and SJ_height_, no significant changes could be determined in CMJ_height_ and DJ_height_, CT and RSI in response to stretching. However, since there was a significant decrease in CMJ performance in the rPA, it can be hypothesized that 10 min of daily stretch seems to be sufficient to reduce decreases in sport-specific performance (CMJ) or even prevent them (SJ). In DJ_height_, DJct and DJRSI, no significant change in performance was detected in IG and rPA. The lack of significant differences in DJ performance due to stretching may be attributable to short contact times of <250 ms, so a minor influence of MSt, but a higher impact of the stretch-shortening cycle could be hypothesized. Stretching seems to induce significant changes in passive properties showing decreased muscle stiffness [35,48]. In contrast, high stiffness seems to be correlated with better usage of the SSC, leading to higher DJ performance via lower DJ_ct_ [49,50]. Hence, a reduction of the RSI in DJ performance after stretching seems not very surprising. In contrast, since in the SJ the concentric movement is exclusively tested, a major influence of MSt on the performance can be assumed. Correlations found between changes in MSt and jumping performance were not statistically significant, which might be attributed to the comparatively small impact of the plantar flexors MSt in jumping performance, as a greater influence of MSt in the upper legs can be assumed [51,52]). Consequently, increases in jumping performance could mainly be attributed to factors other than the increases in plantar flexors MSt. According to a previous study by Warneke et al. [53], correlation coefficients with r = 0.27–0.46 showed a weak influence of plantar flexors MSt on jumping performances in SJ and CMJ. 

### 4.1. Limitations

RSI and CT showed significant pre-test differences, although other parameters seemed to be more balanced. To reduce this problem, values were transformed to 100% in the pre-test to focus on differences between changes due to the intervention. However, as this procedure differs from other statistics used in this study, comparability seems limited. Furthermore, sex was not balanced, with more male participants. Furthermore, the power analysis performed by G-Power showed a power for the effects of the intervention on MSt with 78.71%. Thus, further studies should include a higher number of participants, which was not possible in the current study, as not all participants were willing to interrupt their daily training routines, even when the semester was finished. In addition, further studies should use randomized trials, since in the present study, participants were divided into CG and IG based on their willingness to participate. Figure 2 provides the individual courses for jumping performance from pre- to post-test, showing an inconsistent effect, which may be attributed to the minor involvement of the calf muscle in jumping performance compared with the quadriceps, underpinning the need for a higher sample size in further studies or extending the intervention protocol to stretch the quadriceps as well. The results showed significantly worse contact times for the DJ of the rPA group, which cannot be explained by data evaluation or the allocation of participants since, in most of the other parameters, no significant baseline difference could be observed.

### 4.2. Practical Applications

In summary, this study shows that 10 min of daily stretching appears to be sufficient to counteract an inactivity-induced decline in strength and, in some cases, in explosive strength performance in healthy participants. Therefore, during periods of limited mobility, 10 min of daily exercise can be recommended for healthy individuals to maintain strength and rapid strength performances and to counteract inactivity-related decreases in strength and flexibility in the calf muscles and jumping performance in listed tests. Further research using longer daily stretching durations or longer intervention periods is required to add, for example, the quadriceps and hamstrings to the intervention protocol.

## 5. Conclusions

Based on the results, 10 min of stretching can be seen as an effective home-based training program to counteract significant decreases in strength, flexibility, and jumping performance in response to a reduced level of physical activity. Using 10 min of daily stretching led to only small to moderate effect size increases in MSt and ROM. This study advances the understanding of the effects of stretch-induced adaptations on jump performance.

## Figures and Tables

**Table 1:** Characteristics of test subjects.

Group	Number (n)	Age (in Years)	Height (in cm)	Weight (in kg)
total	35 (f = 13, m = 22)	25.4 ± 2.9	178.7 ± 9.3	74.7 ± 14.0
IG	17 (f = 6, m = 11)	25.8 ± 3.5	177.8 ± 8.4	76.7 ± 14.2
rPA	18(f = 7, m = 11)	24.9 ± 2.1	179.4 ± 10.2	72.8 ± 13.9

IG = intervention group, CG = control group.

**Table 2:** Intraclass correlation coefficients with 95% confidence interval, and 95% confidence interval of the included parameter.

Parameter	ICC (95% CI)	CV (95% CI)
MVC90	0.996 (0.991–0.998)	1.01% (0.87–1.31)
KtW	0.990 (0.985–0.993)	1.13% (1.03–1.31)
SJ_height_	0.899 (0.856–0.922)	2.27% (1.94–2.42)
CMJ_height_	0.90 (0.88–0.923)	1.98% (1.23–2.11)
DJ_height_	0.86 (0.81–0.892)	2.54% (2.29–2.86)
DJ_ct_	0.85 (0.84–0.89)	2.47% (2.18–2.72)

MVC90 = maximal voluntary contraction in the plantar flexors with a 90° knee angle, KtW = knee-to-wall test, SJ = squat jump, CMJ = countermovement jump, DJ = drop jump, height = jumping height, ct = contact time.

**Table 3:** Provides descriptive statistics, percentage increases, and time effect and time × group interaction effects for DJ_height_ and contact time. Data presented as means ± SD.

Group	Pre-Test	Post-Test	% Increase	Time Effect	Time × Group Effect
IG DJ_height_ in cm	29.5 ± 6.1	30.9 ± 6.1	+5.9, *p* = 0.059	*p* = 0.328 F_(1.32)_ = 0.96 ƞ^2^ = 0.03	*p* = 0.169 F_(1.32)_ = 1.97 ƞ^2^ = 0.06
rPA DJ_height_ in cm	29.6 ± 8.0	29.4 ± 6.5	+0.7, *p* = 0.383		
IG DJ_ct_ in cm/s	0.18 ± 0.41	0.19 ± 0.03	+5.1, *p* = 0.081	*p* = 0.328 F_(1.32)_ = 0.96 ƞ^2^ = 0.03	*p* = 0.169 F_(1.32)_ = 1.97 ƞ^2^ = 0.06
rPA DJ_ct_ in cm/s	0.24 ± 0.67	0.24 ± 0.06	+0.08, *p* = 0.49		

DJ_height_ = drop jump jumping height, DJ_ct_ = contact time in the drop jump, rPA = reduced physical activity group, IG = intervention group.

**Table 4:** Provides descriptive statistics, percentage increases, and time effect and time × group interaction effects for ROM measured via KtW, data presented as means ± SD.

Group	Pre-Test (in cm)	Post-Test (in cm)	% Increase	Time Effect	Time × Group Effect
IG KtWR	11.2 ± 3.2	12.7 ± 3.6	+14.1, d = 0.44, *p* < 0.001 *	*p* < 0.001 F_(1.32)_ = 17.101 ƞ^2^ = 0.34	*p* < 0.001 F_(1.32)_ = 45.668 ƞ^2^ = 0.58
rPA KtWR	10.6 ± 2.7	10.3 ± 3.0	−3.8, d = −0.11, *p* = 0.046 *		
IG KtWL	11.5 ± 3.6	12.7 ± 3.7	+12.04, d = 0.33, *p* < 0.001 *	*p* < 0.001 F_(1.32)_ = 22.53 ƞ^2^ = 0.41	*p* < 0.001 F_(1.32)_ = 26.44 ƞ^2^ = 0.45
rPA KtWL	10.8 ± 2.6	10.8 ± 2.8	−0.6, *p* = 0.38		

IG = intervention group, rPA = reduced physical activity group, KtW = knee-to-wall test, * = significantly different from pre- to post-test.

## Data Availability

Data can be provided from corresponding authors due to reasonable request.
